# Alcohol mixed with energy drinks among university students in Poland: prevalence, determinants, and health implications

**DOI:** 10.3389/fpsyt.2026.1772697

**Published:** 2026-03-10

**Authors:** Paulina Mularczyk-Tomczewska, Tytus Koweszko, Mariusz Gujski, Łukasz Czyżewski, Andrzej Silczuk

**Affiliations:** 1 Department of Public Health, Faculty of Health Sciences, Medical University of Warsaw, Warsaw, Poland; 2 Department of Community Psychiatry, Faculty of Health Sciences, Medical University of Warsaw, Warsaw, Poland; 3 Department of Geriatric Nursing, Faculty of Health Sciences, Medical University of Warsaw, Warsaw, Poland

**Keywords:** adolescent health, AMED, caffeine and taurine, energy drinks, public health protection, sociodemographic factors

## Abstract

**Background:**

Alcohol mixed with energy drinks (AmED) has emerged as a public health concern, particularly among young adults. Evidence shows that combining energy drinks (EDs) with alcohol can mask ethanol’s sedative effects, promoting higher intake and risky behaviors.

**Methods:**

A cross-sectional online survey was conducted among university students in the Mazovia region of Poland (July 2025). An anonymous questionnaire assessed sociodemographic characteristics, EDs consumption, AmED use, co-use of psychoactive substances, consumption contexts, and adverse effects. Associations were analyzed using chi-square tests and logistic regression.

**Results:**

A total of 871 students (mean age = 22.1 ± 3.05 years; 73.2% women) participated. One in four (25.5%) reported at least occasional AmED use. Logistic regression showed that a later age of first energy drink consumption was associated with a higher likelihood of AmED (OR = 1.34; 95% CI: 1.14–1.56; p<0.001). Gender was the only significant sociodemographic predictor. Men were more likely to report co-consumption (OR = 1.37; 95% CI: 1.00–1.86; p < 0.05). Over half did not mix energy drinks with other substances; when they did, nicotine (e-cigarettes) and caffeine (coffee) were the most common. The main AmED social contexts were parties and studying, differing by gender. About one-third (36.7%) of AmED users experienced reported unspecified self-reported adverse effects.

**Conclusions:**

AmED consumption is common among university students from the Mazovia region of Poland and appears to be associated with contextual factors, while age of ED initiation showed an ordinal association with the likelihood of AmED use. These findings may inform prevention strategies addressing social drinking contexts and promoting responsible attitudes toward stimulant–alcohol co-use among young adults.

## 1 Introduction

In recent decades, significant changes have been observed in the consumption patterns of non-alcoholic and alcoholic beverages, which have a substantial impact on public health. One of the most prominent trends is the rapid increase in the popularity of energy drinks (EDs) and the practice of mixing them with alcohol (1). Mixing alcohol with EDs (Alcohol mixed with Energy Drink, AmED) has raised growing concern due to its potentially serious health and social consequences (2–4). This phenomenon, particularly common among adolescents and young adults, has become an important public health challenge.

The introduction of Red Bull to the market in 1987 is considered the beginning of the dynamic development of the EDs segment (5, 6). These products quickly gained immense popularity, especially among young people, and their widespread use has become a challenge for public health (7–9). According to the European Commission, the term “energy drink” has no formal legal definition and is merely a marketing term used to describe products containing various substances with stimulating effects on the body. In accordance with the definition of the U.S. Food and Drug Administration (FDA), EDs constitute a group of liquid products whose characteristic ingredient is caffeine, often supplemented with other bioactive substances. They usually contain large amounts of caffeine, simple sugars, and additives such as guarana, taurine, and L-carnitine. The composition of EDs mainly includes caffeine and its natural equivalents (e.g., guarana, yerba mate), B vitamins, amino acids such as taurine and carnitine, as well as sugars, sweeteners, and plant extracts (e.g., ginseng, green tea) (10–12). The compounds present in these beverages can increase energy, alertness, and concentration but at the same time contribute to increased blood pressure, accelerated heart rate, and intensified respiration (13, 14).

Unlike many other psychoactive substances, EDs and so-called energy shots are legally available even to minors (15) (although in Poland, since 2024, their sale to individuals under 18 years of age has been prohibited) (7, 9, 16). Caffeine, the main active ingredient in these beverages, does not cause a sudden release of dopamine and therefore is not classified as a typical stimulant drug. Nevertheless, consuming high doses may lead to the development of dependence, withdrawal symptoms, and dangerous interactions with other substances (15).

The practice of AmED includes both ready-to-drink alcoholic–energy beverages and the self-mixing of alcohol with EDs (17). AmED allows consumers to maintain a sense of stimulation while simultaneously reducing the perception of alcohol’s sedative effects. The presence of multiple bioactive ingredients in EDs makes their combination with ethanol a potential health hazard (18–20). The first warnings about the harmful effects of AmED emerged after numerous cases of hospitalizations of young individuals due to severe intoxication. Research confirms that AmED is associated with increased alcohol consumption, more frequent risk-taking behaviors, and more severe health consequences (21).

It has been demonstrated that caffeine combined with alcohol does not reduce its effects on the body; instead, it promotes excessive drinking and may increase the risk of complications, including alcohol-related diseases and even premature death. Among the potential consequences are hypertension, cardiac arrhythmias, and dehydration. Individuals who consume AmED are more likely to engage in episodes of heavy drinking (binge drinking), risky sexual behaviors, sustain injuries, and drive under the influence of alcohol (22).

In the United States, in November 2010, the U.S. Food and Drug Administration (FDA) deemed the addition of caffeine to alcoholic beverages unsafe and banned the sale of such products. However, the problem of self-mixing alcohol with EDs persists, as approximately 20% of students report engaging in this practice (15). Similarly, the results of the 2019 European ESPAD study, which included over 49,000 students from 17 countries, indicate that 33.9% of adolescents had consumed AmED within the past year, with a higher prevalence among boys compared to girls (17).

The aim of the present study was to explore patterns of ED consumption and their combination with alcohol and other psychoactive substances among university students in the Mazovia region, as well as to identify circumstances favoring such co-use, the occurrence of adverse effects, and sociodemographic factors associated with these behaviors.

## 2 Materials and methods

### 2.1 Study design

This descriptive cross-sectional study was carried out between 20 and 31 July 2025 among students of higher education institutions located in the Mazovia region of Poland. Data collection was performed through an anonymous online questionnaire shared across various social media channels, such as university groups and student discussion forums. Participation was entirely voluntary; before starting the survey, each respondent provided electronic informed consent after reviewing information about the study’s purpose, the guarantee of anonymity, and the option to withdraw at any stage. To be eligible, individuals had to be currently enrolled at a university or other higher education institution in Mazovia during the study period.

The questionnaire addressed sociodemographic characteristics, patterns of combining EDs with alcohol and other psychoactive substances, contexts and circumstances of such consumption, experienced adverse effects, and the age at first EDs intake. The study protocol was reviewed and approved by the Bioethics Committee at the Medical University of Warsaw, Poland (decision number AKBE/139/2025, 12.05.2025).

### 2.2 Questionnaire and study measures

The study questionnaire was designed after reviewing available literature on EDs consumption and co-use with alcohol and other psychoactive substances. It included sections addressing participants’ sociodemographic characteristics; patterns of combining EDs with alcohol and other substances; circumstances and contexts of such use (e.g., social events, studying, stressful situations); age at first consumption of EDs; and possible adverse effects experienced after co-consumption. Respondents were also asked about additional behaviors, including the use of tobacco, e-cigarettes, caffeinated beverages, and other stimulants.

Key questionnaire items relevant to the primary outcomes were as follows: participants were asked, “Do you mix energy drinks with alcohol (e.g., vodka)?” (response options: definitely no/rather no/hard to say/rather yes/definitely yes), and “Have you ever experienced negative effects after combining an energy drink with alcohol or another substance?” (response options: definitely no/rather no/hard to say/rather yes/definitely yes). Age at first energy drink consumption was assessed with the item: “When did you first consume an energy drink?” (before the age of 15/between 15 and 18 years/after the age of 18/I do not remember/never).

Before data collection, the questionnaire was pretested with a small pilot group of students (n = 30) not included in the final sample to ensure clarity and to estimate completion time. Minor adjustments were made to refine wording and improve comprehensibility. Sociodemographic data included gender, age, place of residence, type and level of study program, mode of study (full-time or part-time), and self-assessed financial status.

The AmED use variable was assessed using a self-report item capturing general mixing behavior (alcohol combined with energy drinks) without specifying a defined time frame or consumption frequency; therefore, the measure reflects self-reported AmED use within an unspecified time frame. Age at first energy drink use was entered into the regression model as an ordinal variable to assess an overall trend between earlier and later initiation, acknowledging that this parameterization assumes monotonicity and equal spacing across categories and may simplify potential non-linear differences between exposure groups.

### 2.3 Sample size and recruitment

Recruitment was conducted using convenience sampling by sharing an open survey link. To reduce the risk of self-selection bias associated with this approach, the questionnaire was distributed across a wide range of student groups and academic disciplines. Sample size estimation using G*Power for the chi-square test (medium effect size, α = 0.05, power = 0.80) indicated that a minimum of 384 participants was needed. The study ultimately included 871 respondents, which provided robust statistical power.

### 2.4 Statistical analysis

Statistical analyses were performed using the *Statistica 13.3* software package with the Plus module (23), under a license held by the Medical University of Warsaw (MUW). The selection of analytical methods was adapted to the nature of the data and the level of measurement of the variables. Prior to selecting the statistical procedures, assumptions appropriate for each method (including data distribution, scale of measurement, and group sizes) were evaluated to ensure the suitability of the applied tests.

To assess the relationships between categorical and ordinal variables, the median test was applied, enabling comparison of the distributions of the dependent variable across groups defined by factors such as gender, place of residence, financial situation, and year of study. This test made it possible to determine whether the frequency of reported negative effects of combining energy drinks with other substances differed significantly between the analyzed groups. As an additional nonparametric approach, the median test was used to compare the distribution of the ordinal outcome across categorical groups when parametric assumptions were not met, complementing the Kruskal–Wallis and Mann–Whitney U tests.

For comparisons involving more than two groups, and in the absence of assumptions of normality, the Kruskal–Wallis test was used to assess differences in the distribution of the dependent variable in a nonparametric framework. For comparisons between two independent groups, the Mann–Whitney U test was employed to evaluate the significance of differences in the distribution of an ordinal variable without requiring parametric assumptions. To identify predictors of specific behaviors, logistic regression (logit model) was applied, allowing the modeling of the probability of a given phenomenon (e.g., combining energy drinks with alcohol) based on independent variables. The regressions included gender limited to two categories (female and male), which enhanced the methodological rigor of the analysis. For each model, regression coefficients, odds ratios (OR), 95% confidence intervals, Wald test statistics, and the chi-square value for the overall model were reported.

All results were interpreted at the assumed level of significance of p < 0.05, taking into account the data distribution, sample sizes within categories, and the adequacy of the applied methods in relation to the level of measurement of the variables.

## 3 Results

### 3.1 Characteristics of the study group

A total of 871 students participated in the study (mean age 22.1 ± 3.05 years; 73.2% women). All respondents were residents of the Mazovia region, with the majority living in large urban areas. Detailed sociodemographic characteristics of the study participants are presented in **Table 1**.

**Table 1:** Sociodemographic characteristics of the study group.

Category	M	SD	Min-max
Age	22,12457	3.05	18-45
		**N**	**%**
Sex	Female	638	73.2
	Male	220	25.3
	Other	7	0.8
	Prefer not to disclose	6	0.7
Place of residence	Countryside (village)	89	10.2
	Small town (up to 20,000)	47	5.4
	Medium-sized city (20,000–100,000)	95	10.9
	Large city (100,000–500,000)	58	6.7
	Metropolitan city (>500,000)	582	66.8
Financial situation	Very good	250	28.7
	Good	517	59.4
	Difficult to say	86	9.9
	Poor	16	1.9
	Very poor	2	0.2
Type of study program	Bachelor’s degree	328	37.7
	Master’s degree	185	21.2
	Integrated Master’s program	358	41.1
Mode of study	Full-time	743	85.3
	Part-time	128	14.7
Year of study	I	201	23.1
	II	227	26.2
	III	200	23.0
	IV	136	15.6
	V	82	9.4
	VI	25	2.9

### 3.2 Age at first consumption of EDs

**Figure 1:**
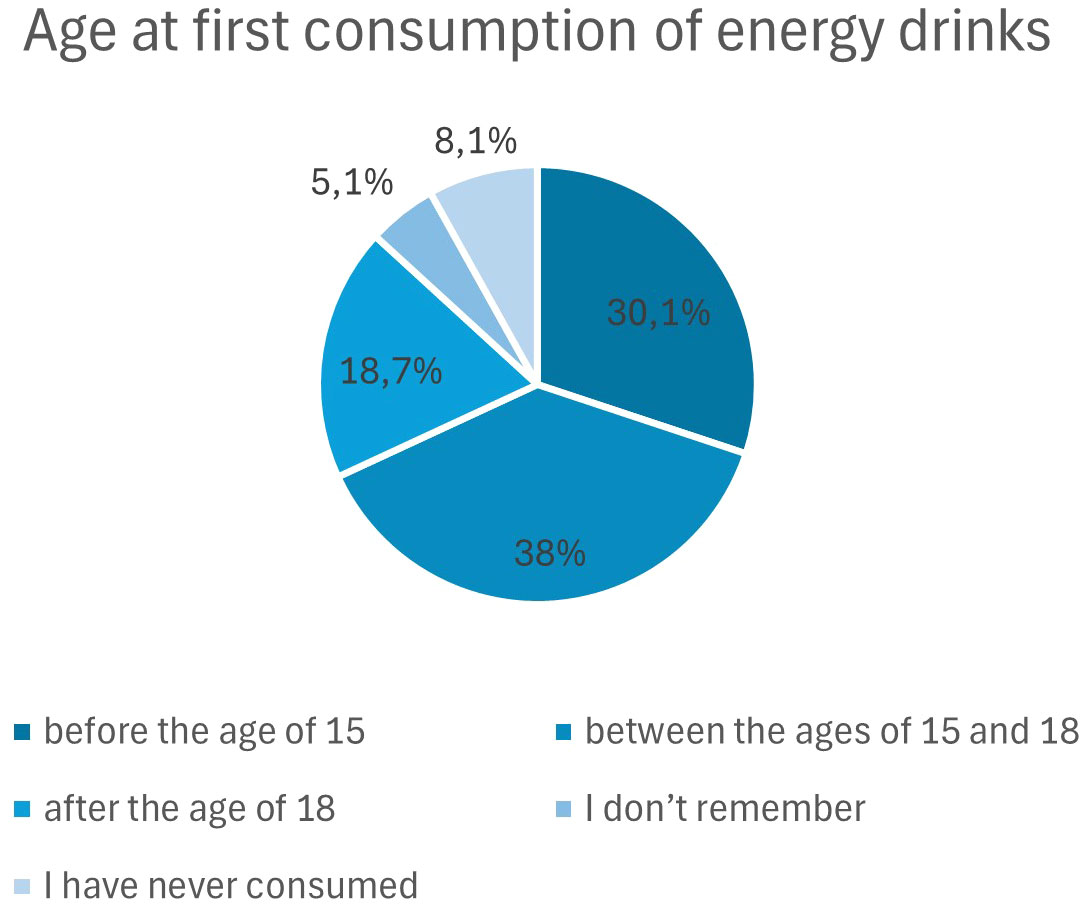
Age at first consumption of energy drinks among study participants.

Most respondents reported first consuming energy drinks between the ages of 15 and 18 (38.0%). Nearly one-third (30.1%) initiated use before the age of 15, whereas 18.7% reported first consumption after the age of 18. A small proportion did not remember their age at first use (5.1%), and 8.1% had never consumed energy drinks. Overall, these findings indicate that initial exposure to energy drinks occurs primarily during adolescence, with a substantial proportion beginning use before age 15 (**Figure 1**). Logistic regression analysis was conducted to examine whether age at first energy drink consumption was associated with the likelihood of mixing energy drinks with alcohol. Responses “I don’t remember” were treated as missing and excluded from the analysis.

As shown in **Table 2**, age at first energy drink consumption was significantly associated with the likelihood of mixing energy drinks with alcohol, indicating an overall ordinal trend toward higher odds of co-consumption with later initiation.

**Table 2:** Crude and adjusted logistic regression analyses of predictors of mixing alcohol with energy drinks.

Factor	Crude OR (95% CI)	p	Adjusted OR (95% CI)	p
Gender	1.37 (1.00–1.86)	0.046	1.41 (1.02–1.94)	0.036
Place of residence	1.09 (0.98–1.20)	0.095	1.07 (0.97–1.19)	0.19
Financial situation	1.04 (0.85–1.27)	0.689	1.02 (0.83–1.25)	0.87
Year of study	0.95 (0.86–1.04)	0.26	0.91 (0.82–1.00)	0.06
Age at first consumption of an energy drink	1.34 (1.14–1.56)	<0.001	1.35 (1.15–1.59)	<0.001

### 3.3 Combining alcohol and EDs

**Figure 2:**
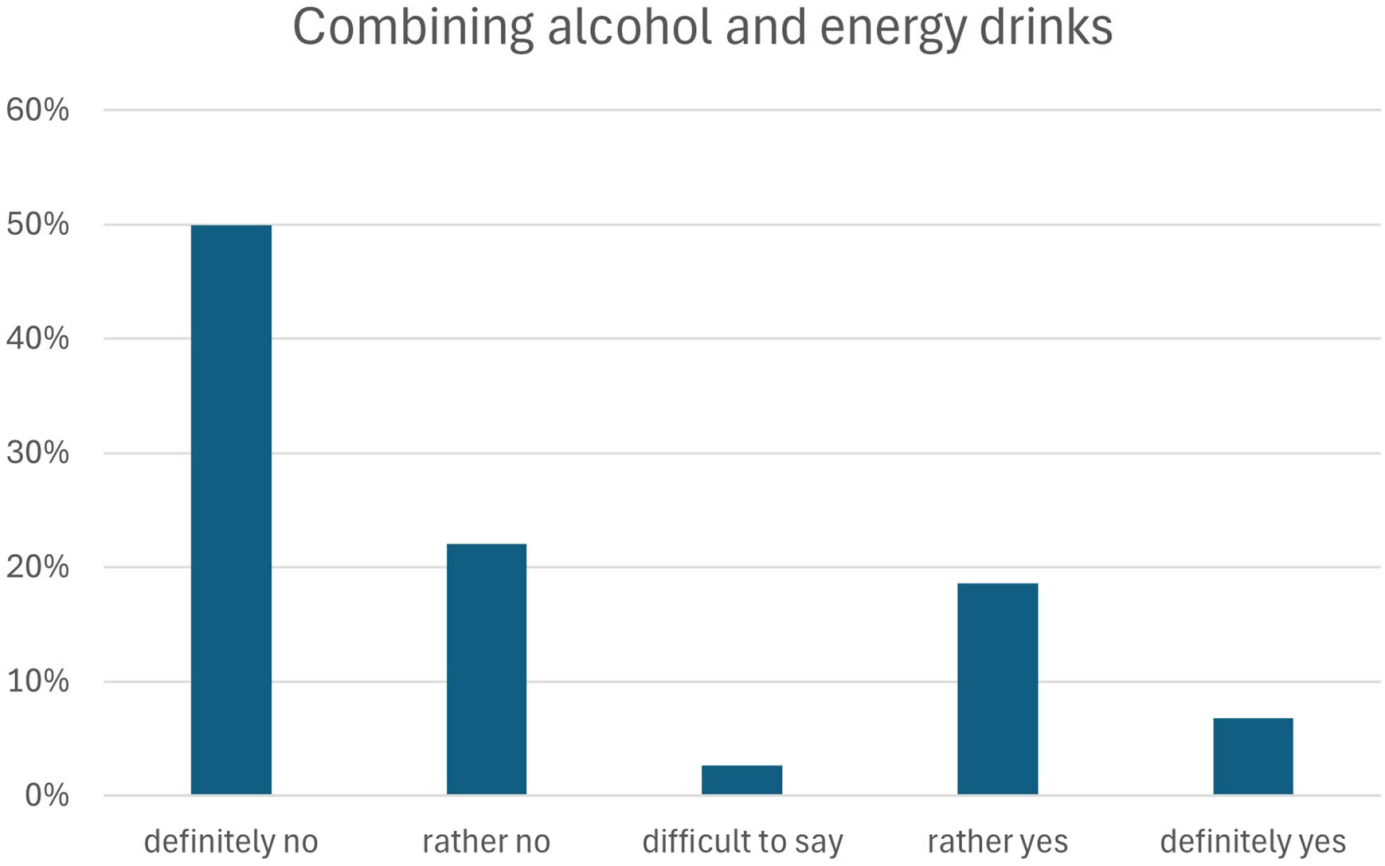
Frequency of combining alcohol and energy drinks among study participants.

Most respondents reported that they do not combine alcohol with energy drinks: 49.9% answered “definitely no” and 22.0% “rather no,” totaling 72.8% of participants. Occasional co-consumption was reported by 25.5% (“rather yes” 18.6%; “definitely yes” 6.8%), while 2.6% were uncertain (**Figure 2**).

**Table 2** presents crude and adjusted logistic regression models examining predictors of mixing alcohol with energy drinks. The multivariable logistic regression model was statistically significant, χ²(5) = 22.619, p = 0.0004, indicating that the set of predictors reliably distinguished between individuals who mix alcohol and energy drinks and those who do not. Male sex and later age at first energy drink consumption were associated with higher odds of mixing alcohol with energy drinks, whereas higher year of study showed a borderline inverse association. Place of residence and financial situation were not significantly associated with the outcome.

### 3.4 Co-consumption of EDs with other substances

**Figure 3:**
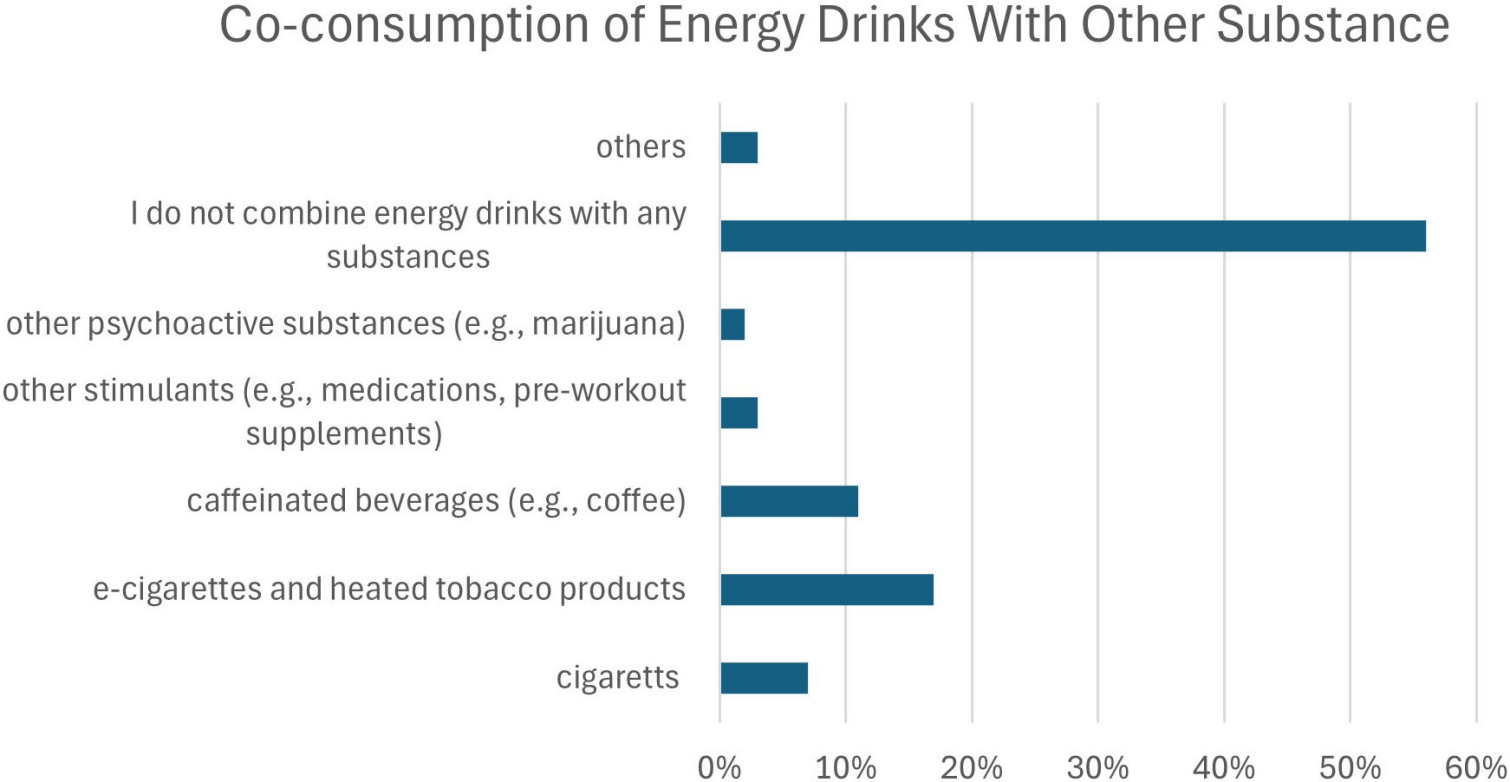
Co-consumption of energy drinks with other substances among study participants.

More than half of respondents (56.7%) reported that they do not combine energy drinks with any other substances. Among those reporting co-use, the most common combinations involved e-cigarettes or heated tobacco products (17.1%) and caffeinated beverages such as coffee (10.8%), followed by cigarettes (7.0%), other stimulants (3.0%), and psychoactive substances (2.4%). Detailed distributions are presented in **Figure 3**.

Logistic regression models examining sociodemographic predictors of combining energy drinks with other substances are presented in **Table 3**. Place of residence and poorer financial situation were associated with higher odds of combining energy drinks with cigarettes, whereas higher year of study was associated with lower odds of this behavior. Male sex was associated with lower odds of combining energy drinks with e-cigarettes and coffee but higher odds of combining energy drinks with psychoactive substances. Most other associations were not statistically significant, and no sociodemographic predictors were identified for combining energy drinks with other stimulants.

**Table 3:** Sociodemographic predictors of co-use of energy drinks with selected substances (adjusted logistic regression).

Outcome	Factor	OR 95% CI	P
Cigarettes	Male sex	0.70 (0.47–1.04)	0.079
	Place of residence	1.17 (1.03–1.33)	0.017
	Financial situation	1.30 (1.03–1.65)	0.030
	Year of study	0.87 (0.77–0.99)	0.030
E-cigarettes	Male sex	0.60 (0.39–0.92)	0.018
	Place of residence	1.11 (0.98–1.27)	0.108
	Financial situation	1.27 (0.99–1.63)	0.059
	Year of study	0.90 (0.79–1.02)	0.113
Coffee	Male sex	0.44 (0.26–0.77)	0.004
	Place of residence	1.08 (0.93–1.26)	0.312
	Financial situation	1.37 (1.02–1.83)	0.035
	Year of study	0.96 (0.83–1.11)	0.583
Stimulants	Male sex	0.92 (0.41–2.08)	0.842
	Place of residence	1.13 (0.85–1.50)	0.403
	Financial situation	0.83 (0.49–1.41)	0.485
	Year of study	0.98 (0.76–1.27)	0.893
Psychoactive substances	Male sex	2.99 (1.12–7.96)	0.028
	Place of residence	1.60 (0.89–2.88)	0.116
	Financial situation	0.57 (0.26–1.25)	0.159
	Year of study	0.82 (0.56–1.19)	0.299

### 3.5 Most frequent situations of combining EDs with alcohol or other psychoactive substances

**Figure 4:**
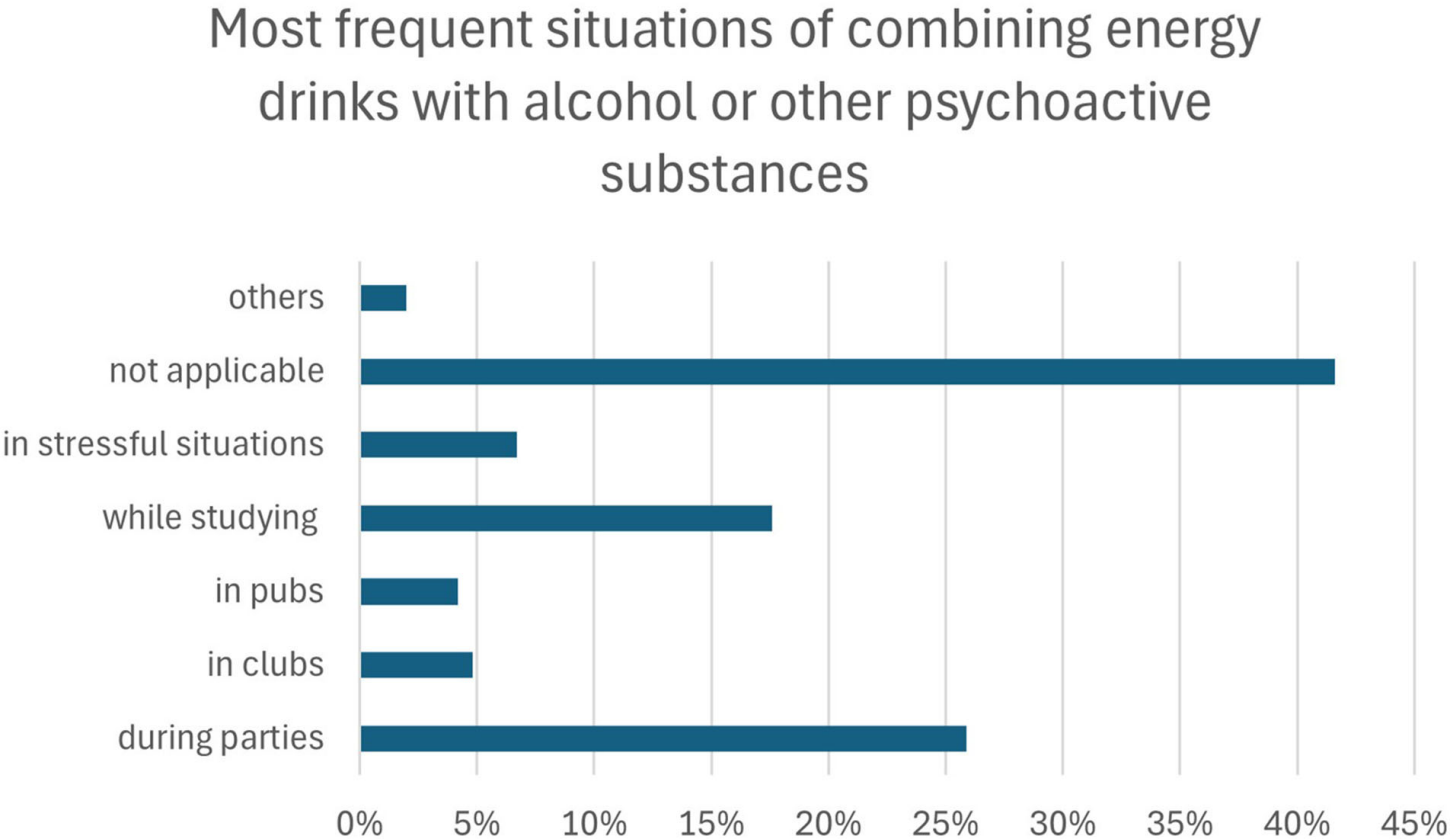
Situational contexts of co-consumption of energy drinks with alcohol or other psychoactive substances.

The most frequently reported context of combining energy drinks with alcohol or other psychoactive substances was “not applicable” (41.6%), indicating no engagement in such behavior. Among those reporting co-use, the most common settings were parties (25.9%) and studying (17.6%), followed by stressful situations (6.7%), clubs (4.8%), and pubs (4.2%); other contexts were rarely indicated (2.0%) (**Figure 4**).

Logistic regression analyses of sociodemographic predictors of reported contexts are summarized in **Table 4**. Gender was the most consistent predictor: men were more likely to report parties and less likely to report studying or stress as contexts of co-consumption. No significant predictors were identified for clubs, and other variables showed limited or inconsistent associations across models. The model for pubs also reached statistical significance; however, none of the individual predictors met the conventional significance threshold, although year of study showed a borderline association (p = 0.054).

**Table 4:** Sociodemographic predictors of reported circumstances of combining energy drinks with alcohol or other substances (adjusted logistic regression).

Context (outcome)	Factor	OR 95% CI	P
Parties	Male sex	1.59 (1.15–2.20)	0.006
	Place of residence	1.03 (0.92–1.15)	0.607
	Financial situation	1.17 (0.94–1.45)	0.164
	Year of study	0.96 (0.86–1.07)	0.489
Clubs	Male sex	1.20 (0.69–2.11)	0.521
	Place of residence	1.12 (0.92–1.38)	0.262
	Financial situation	0.95 (0.65–1.39)	0.804
	Year of study	1.00 (0.85–1.17)	0.962
Pubs	Male sex	1.81(0.94–3.50)	0.077
	Place of residence	1.21 (0.91–1.59)	0.184
	Financial situation	1.22 (0.79–1.90)	0.370
	Year of study	1.24 (1.00–1.55)	0.054
Studying	Male sex	0.59 (0.38–0.90)	0.015
	Place of residence	1.02 (0.90–1.16)	0.756
	Financial situation	1.09 (0.85–1.41)	0.487
	Year of study	0.93 (0.82–1.06)	0.261
Stress	Male sex	0.38 (0.18–0.82)	0.014
	Place of residence	0.99 (0.82–1.19)	0.910
	Financial situation	1.03 (0.70–1.52)	0.879
	Year of study	0.95 (0.79–1.15)	0.620

The models predicting reporting study-related situations and stress showed trends toward overall significance, and in both cases, gender was the only significant predictor, with men less likely to report these contexts (study: OR = 0.59; p = 0.015; stress: OR = 0.38; p = 0.014). No statistically significant predictors were identified for reporting clubs as the context of co-consumption. Overall, place of residence, financial situation, and year of study were not consistently associated with the analyzed circumstances.

### 3.6 Experience of negative effects after combining EDs with alcohol or other substances

**Figure 5:**
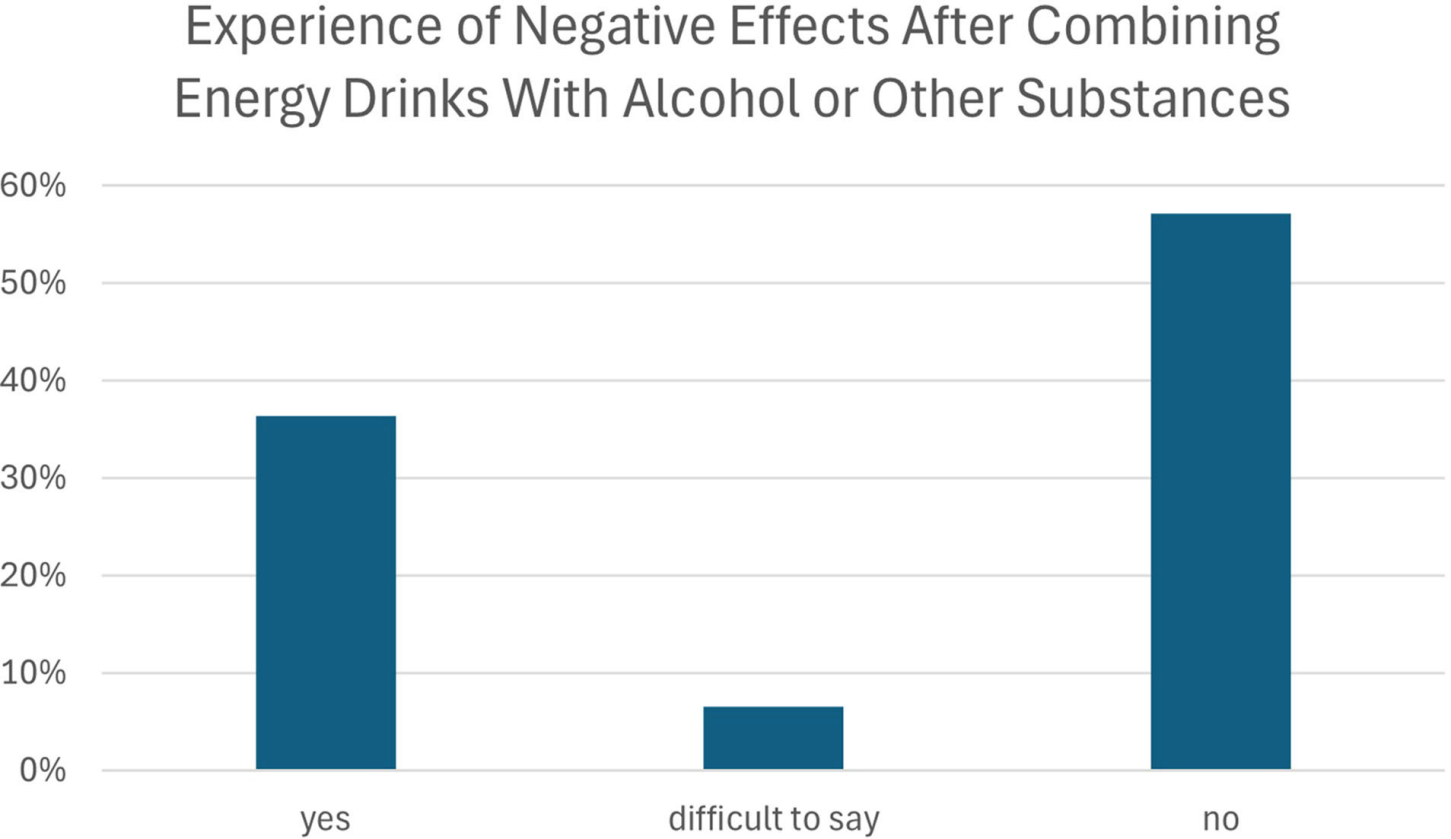
Self-reported negative effects of co-consuming energy drinks with alcohol or other substances.

Among respondents who reported combining EDs with alcohol or other psychoactive substances, more than one-third (36.4%) reported unspecified self-reported negative effects. Over half of participants (57.1%) stated that they had not noticed any adverse outcomes, while 6.5% were uncertain or unable to clearly assess whether such effects had occurred (**Figure 5**). The reported adverse effects were assessed using a general self-report measure without specification of symptom type or severity.

Associations between sociodemographic variables and self-reported negative effects following the combined use of energy drinks with alcohol or other substances were examined using non-parametric tests. No statistically significant differences were observed for place of residence, financial situation, or year of study (median tests), and gender differences were also not significant (Kruskal–Wallis test; all p > 0.05).

## 4 Discussion

In the present study, patterns of EDs consumption and their combination with alcohol and other psychoactive substances were analyzed among university students from the Mazovia region.

Although most respondents declared that they do not mix EDs with alcohol, the proportion of students engaging in such behavior remains noticeable, as approximately one quarter of the participants admitted to at least occasional mixing of these beverages with alcohol. A similar proportion was reported in studies conducted in the United States, where 27% of students declared consuming alcohol mixed with EDs within the past 30 days (18).

Similar observations have also been reported among younger age groups. For example, a study of Italian adolescents found that approximately half of them mix EDs with alcohol, which may increase the risk of health-threatening behaviors (24). Findings from a Portuguese study indicate a high prevalence of EDs consumption among adolescents, with nearly 40% reporting mixing them with alcohol, which confirms the scale of the problem already in younger age groups and highlights the need for early prevention (25). These results are consistent with observations that individuals who consume EDs are more likely to engage in other unhealthy behaviors (26, 27). Together, these findings suggest that the phenomenon is widespread and represents a public health concern across different age groups and European countries (28).

The results obtained align with the broader European context. Data from the 2019 ESPAD survey, covering more than 49,000 students from 17 countries, indicate that 33.9% of adolescents consumed AmED in the past year, with this proportion being higher among boys (37.3%) than girls (30.6%) (17). Analysis of consumption patterns in international studies has also shown that nearly half of young people who have tried AmED report doing so only once or twice a year; however, there are also groups of users who consume these mixtures much more frequently, and frequent AmED intake is associated with a higher risk of psychoactive substance use and engagement in risky behaviors, such as binge drinking, fighting, or risky sexual activity (29).

Brazilian studies have shown that students who consume alcohol mixed with EDs are more likely to engage in risky drinking patterns and unsafe driving behaviors, such as speeding or driving after episodes of heavy alcohol consumption. These findings suggest that combining alcohol with EDs may increase young adults’ vulnerability to dangerous behaviors (30).

The present results indicate a significant, though weak, association between gender and the practice of mixing EDs with alcohol. Men were more likely to report such behaviors, which is consistent with previous observations showing a higher prevalence of EDs and AmED consumption among boys and young men (29, 31, 32).

The obtained results revealed that a later age of first EDs consumption was associated with a higher likelihood of AmED, which contrasts with previous findings indicating that earlier initiation typically increases the risk of alcohol-related behaviors. This reverse pattern may reflect the heterogeneity within the student population. While many respondents reported first consuming EDs during adolescence for functional purposes such as studying or improving concentration, those who initiated use later often after the age of 18 were more likely to do this in social or alcohol-related contexts. In this group, the first use of EDs may have coincided with, or reflected, their first experience of AmED. In some European countries, legal regulations are already present to support ED use prevention in minors (33). These interpretations should be considered hypothesis-generating given the cross-sectional study design.

This discrepancy from studies such as the Canadian COMPASS Host Study likely stems from differences in the behaviors examined: early alcohol initiation tends to reflect adolescent experimentation, whereas later initiation of EDs use among university students often occurs within social and recreational contexts involving alcohol (34). It is therefore plausible that, in this age group, exposure to alcohol precedes or coincides with the first consumption of EDs. In such cases, the self-reported age of first use may reflect not only chronological age but also the situational context of this experience often indicating the first exposure to AmED rather than to EDs themselves.

The findings showing co-occurrence of mixing EDs not only with alcohol but also with other psychoactive substances align with observations from Italian researchers, who demonstrated a significant association between ED and AmED consumption and the use of cannabis, other illicit drugs, and daily tobacco smoking. These results suggest that EDs consumption among adolescents and young adults is often part of broader polysubstance risk behaviors, posing an important challenge for preventive interventions (35). These findings highlight the need for the development of effective prevention strategies (36).

## 5 Limitations of the study

Several limitations of this study should be acknowledged when interpreting the findings. First, the cross-sectional design precludes any causal inferences regarding the relationships between energy drink consumption, alcohol mixing, and associated behaviors; observed associations reflect correlations only and may be influenced by unmeasured confounding factors. Second, data were collected using a self-reported online questionnaire, which introduces the risk of recall bias and social desirability bias, particularly for behaviors related to alcohol and psychoactive substance use. In addition, AmED use was assessed without a specified time frame or information on consumption frequency or intensity, which limits comparability with studies reporting time-bound prevalence estimates and requires cautious interpretation of health implications.

Third, the use of convenience sampling and recruitment through social media channels may have introduced selection bias, potentially overrepresenting more socially active or health-aware students and thereby limiting the representativeness of the sample. The study was also conducted in a single Polish region with a predominance of female respondents, which restricts the generalizability of the results to other geographic areas, academic settings, or more gender-balanced student populations.

Finally, the ordinal coding of age at first energy drink use may oversimplify differences between exposure groups, and future studies should consider modeling this variable using categorical indicator variables with an explicit reference group. Although the questionnaire covered a wide range of consumption contexts and adverse effects, the lack of objective measures such as biomarkers or validated diagnostic instruments limits the precision of exposure and outcome assessment. Future research would benefit from longitudinal designs, probabilistic sampling strategies, and the inclusion of objective or clinically validated measures to better elucidate temporal relationships and strengthen external validity.

## 6 Conclusions

This study indicates that the consumption of EDs and AmED remains prevalent among university students from the Mazovia region of Poland, with a considerable proportion engaging in these behaviors at least occasionally. Among students, EDs may be first introduced in social settings linked to alcohol consumption, suggesting that AmED often represents a component of broader social drinking patterns. The observed co-use of EDs with other psychoactive substances is consistent with the presence of broader polysubstance-related risk behaviors.

These findings may inform targeted prevention strategies addressing not only initiation patterns but also the situational and social contexts of AmED use. Educational programs should focus on increasing awareness of the risks associated with stimulant alcohol combinations and on promoting responsible consumption behaviors among young adults.

## Data Availability

The raw data supporting the conclusions of this article will be made available by the authors, without undue reservation.
